# Genito-Urinary and Syphilitic Diseases

**Published:** 1898-01

**Authors:** Byron H. Daggett

**Affiliations:** Buffalo, N. Y.


### GENITO-URINARY AND SYPHILITIC DISEASES.

CONDUCTED BY BYRON H. DAGGETT, M. D., Buffalo, N. Y.

### INFLAMMATION OF JOINTS IN GONORRHEAL PATIENTS.

E. J. SENN (*Medical Record*, July 31, 1897). The term gonorrheal arthritis indicates the etiology and pathology of the joint complications following gonorrhea, although it is the exception to find the gonococci in the joint's exudate. Gonorrheal rheumatism has been recognised for centuries, but its specified nature was not demonstrated until the discovery of the gonococcus. Even at that time metastases of gonorrheal origin have been sufficiently proven to establish a common derivation. It is claimed that gonorrheal arthritis is not a common complication of that malady, yet in a large number of cases it is erroneously diagnosticated as tuberculosis, rheumatism or some other malady. Its cause is often overlooked, because it occurs in the latter stages of gonorrhea or as a result of chronic posterior urethritis, unrecognised or unappreciated.

Many theories have been proposed to explain the etiology of gonorrheal rheumatism. The discovery of the gonococcus opened a way for the study of the pathology of gonarthritis. Rosalimus and Lewin believed that the specific toxin produced pathologic conditions through reflex action on the vasomotor nerves. Senator explains the cause as the local irritation affecting the trophic nerve fibers. Guyon and Janet believed that the gonococcic ptomaines cause the metastasis. Shuster refers the cause to a combination of syphilis and gonorrhea. Diday believed that the gonorrheal poison causes complications through rheumatic diathesis. Theiry maintains that there is no direct connection between gonorrhea and the rheumatic diathesis. Peter and Bouillard alleged that gonorrheal arthritis is due to rheumatism. Duboc said it was due to a gouty diathesis, because later in life it is often polyarticular. Tommasoli and Hutchinson considered gonorrheal arthritis only articular rheumatism. Garrad said it is due to anemia. Loeb said it is due to the gonorrheal process in the posterior urethra, which acts as an infecting antrum for the passage of the pus microbe into the lymph and blood streams and then to the locus minoris resistantiæ in the joint. Fournier believed gonarthritis due to injury of the urethra and called it urethral rheumatism. Panas stated that catheterisation arthritis is due to infection through erosion, causing kidney congestion from septicemia and consequent uremia and resulting arthritis. Ricard and Bergh thought gonarthritis due only to chronic posterior urethritis. Bond believes the joint complications are caused by inflammation of the prostatic veins and not of the lymphatics, since the lymphatics are affected early in the disease and such lymphatic inflammation is not followed by joint complications. Guerin, Loraine and Lesigne consider gonorrhea a general disease, because it produces remote complications. The local manifestation is a period of incubation and remote complications may not ensue if the disease is arrested at this period.

Gonarthritis is due to the invasion of the gonococcus. Paltauf and Long found gonococci in joint effusions and demonstrated their character by cultivation. Many others have failed to find the specific microbe. Hewes reports two cases in which he found the organism in the blood. Joint inflammation is most often found in young adults and has the appearance of a primary osteoperiostitis. Gonorrheal microbes may cause general infection and arthritis without being germinated in a specific urethritis. Several observers note its following blennorrhea neonatorum. The disease is usually monoarticular and may affect any joint, but most frequently attacks the knee, elbow or wrist. One attack does not render immunity to another. Recrudescence of the urethritis, or a new attack, is followed, almost without exception, by joint complications in cases once having had gonarthritis. Catheterisation is liable to cause exacerbation of joint symptoms. Basset reported a patient who had gonorrhea five times in one year, presumably an auto-infection, followed each time by arthritis.

During the acute inflammation the gonococcus rarely leaves its habitation, the mucous membrane, but after the acute symptoms subside the latent microbe emigrates, in some unknown way, by means of the blood or lymph and settles upon a serous membrane. Upon mucous membrane the gonococcus is a pus microbe; upon serous membrane it plays an inferior rôle and excites plastic inflammation. Burnstead says that gonorrheal rheumatism is essentially a hydrarthrosis and suppuration rarely occurs. If suppuration does occur, it is the result of a mixed infection.

Aubert says the blennorrhagic secretion is alkaline and that the gonococcus thrives on membranes that are alkaline in reaction. The peritoneum and synovial membranes are similar and are forms of mesoblastic tissue, so we may consider, as pointed out by Sinclair, the two different rôles played by gonococci on tissues of different embryological origin.

Gonococcus causes purulent inflammation of mucous membrane and adhesive inflammation in connective tissues.

Kornig classifies gonorrheal arthritis: 1. Hydrops articularis. 2. Hydrops articularis sero-fibrinosus et catarrhalis (Volkman). 3. Empyema of joint. 4. Phlegmon of joint.

The simple hydrops is the mildest and tends to definite healing. The fibrinous form is characterised by flocculent hydrops and a tendency to cause anchylosis. The third form is rare. The phlegmonous type is characterised by a severe form of inflammation, with erosion and disintegration, thickening of capsule and ligaments, phlegmon of connective tissues and often accompanied by inflammation of bursæ and tendon sheaths.

The clinical history of gonorrheal patients and their joint complications are variable. The arthritis may be acute or chronic and seldom appears during the acute stage of gonorrhea, generally about six weeks after the primary symptoms of the urethral inflammation. There is a peculiar swelling of the knee-joint, almost diagnostic, most marked in the upper part of the joint under the vasti muscles. Fluctuation and odema are present and the muscular structures of both the leg and thigh atrophy. The urethral discharge ceases during the acute stage of arthritis and reappears with its decline. Chronic gonarthritis comes on insidiously and gradually fills the joint with a catarrhal transudation, a condition that simulates tubercular hydrops. Before adult life we may find tenderness over the epiphysis, due to primary osseous tuberculosis and secondary synovitis. This diagnostic sign is not present in adults, as the synovial tuberculosis is primary. Diagnosis is made up from the clinical history, macroscopical and microscopical examination of the aspirated joint contents.

Kornig believes that in every case of acute catarrhal inflammation of joints the urethral secretion should be examined and that ninety-nine in every hundred will be found to be gonorrheal in origin if there be a blennorrhagic discharge. Undoubtedly, many cases of gonorrheal hydrops are diagnosticated and treated as tuberculous affections. The prognosis as regards mortality is favorable; as to local functional results is very grave.

The treatment rests upon its etiological basis, which is both active gonococci and predisposing rheumatic diathesis. The salicylates act favorably, but do not shorten the disease. The alkalies, salicylates, salol, lithium carbonate and citrate of potassium are anti-rheumatic and anti-blennorrhagic. Iodide of potassium produces good results. Jullien claims that oil of wintergreen comes nearest to a specific.

R. W. Taylor reports favorable results in twelve cases. Mercurial plaster may be applied to the swollen joints. The original focus should not be lost sight of; it should be actively treated. Ricard and Berg believed that posterior urethritis is necessary for gonarthritis. The joint should be held on a splint to immobilise it and hot fomentations applied during the acute stage; later, absorption of the effusions promoted by mechanical pressure, by external medication or drawn off by puncture. Jullien recommends ichthyol and lanoline, equal parts. The French use vesication in para-articular processes and Kornig applies tincture of iodine in the phlegmonous form; in the hydropic form he taps the joint and injects carbolic acid. When the hydrops are sero-fibrinous, aspiration and injection is especially indicated. Fürtterer, of Chicago, injected half a drachm of the oil of sandal-wood into the affected joint, with brilliant results, in four cases. In cases of empyema, incision and drainage are indicated. Christien advises prompt arthrotomy in all cases, followed by immobilisation and massage. If there are erosions, counter-extension is to be applied. Massage, active and passive motion, should be employed to avoid anchylosis.

---

### TWO HUNDRED AND FIFTY CASES OF GONORRHEA TREATED BY LARGE DOUCHES OF PERMANGANATE OF POTASSIUM.

Dr. Hogge concludes (*Charlotte Medical Journal*): (1) Janet's method is abortion in one-fourth of the cases if seen within forty-eight hours after purulent discharge has appeared. (2) Large douches of permanganate of potassium and lime assure absolute cure in nearly every case of gonorrhea, if treated from the beginning, or if treated rationally until the method is established. (3) This method assures a minimum of complications. Folliculitis and prostatitis are but little influenced by irrigation. (4) It is not necessary to douche the posterior urethra systematically. (5) Weak solutions (1 to 8000 or 1 to 5000) produce the desired result. Strong solutions retard cure and may produce complications.

---

### THE GONORRHEAL INFLAMMATION OF THE JOINTS, BURSÆ AND TENDON SHEATHS.

Neisser states that these maladies may be recognised, although the presence of gonococci may not be demonstrable. The appearance of these complications is variable in time, with different subjects depending upon the rapidity of the invasion of the mucous membrane and deep structures. The older cases more frequently develop joint complications.

These complications occur regardless of the locality of the infection. The joints most exposed to traumatism are most frequently attacked, as the knee in the male and wrist in the female. There is danger of anchylosis from mild attacks, due to contractions of the synovia and the peri-articular tissue. The effusion is usually scanty and sero-fibrinous in character. Rarely, the attack begins with pyemic symptoms and purulent effusion. In such cases the cartilaginous surfaces of the bones are destroyed, causing caries and necrosis. Tendo-vaginitis may occur, isolated or in conjunction with the joint lesion. These conditions in combination point to gonorrheal origin.

Inflammations of the wrist and ankle bursa are more rare, are extremely painful and obstinate, and associated with cicatricial induration. There is a peculiar tissue proliferation in and around the joint. Relapses are very prone to occur as long as the primary infection exists or a new one has been contracted. Local treatment consists of compression, rest, immobilisation, massage, warm baths and Preisnitz bandages.

Effusion and swelling call for puncture and injection of iodoform. In severe cases, drainage is indicated. Mild astringents should be used for treatment of the primary disease. Irritating injections should be avoided lest they induce fresh metastases.

